# Descriptive analysis of routine childhood immunisation timeliness in the Western Cape, South Africa

**DOI:** 10.1016/j.jvacx.2021.100130

**Published:** 2021-12-13

**Authors:** Ntombifuthi Blose, Edina Amponsah-Dacosta, Benjamin M. Kagina, Rudzani Muloiwa

**Affiliations:** a Division of Epidemiology and Biostatistics, School of Public Health and Family Medicine, University of Cape Town, Western Cape, South Africa; b Vaccines for Africa Initiative, School of Public Health and Family Medicine, University of Cape Town, Western Cape, South Africa; c Department of Paediatrics and Child Health, Red Cross War Memorial Children’s Hospital, Western Cape, South Africa

**Keywords:** Childhood immunisation, Vaccine, Vaccine adherence, Vaccine coverage, Vaccine preventable diseases, South Africa

## Abstract

Adherence to recommended age-specific immunisation schedules is critical in ensuring vaccine effectiveness against vaccine preventable diseases (VPDs). There is limited data on immunisation timeliness in sub-Saharan Africa. Therefore, this study assessed the timeliness of age-specific routine childhood immunisation within the Western Cape Province of South Africa. Participant records (N = 709) from a prospective health-facility based study conducted in Cape Town, SA in 2012–2016 were analysed. The outcome measure was receiving age-specific immunisations ≥4 weeks of that recommended for age as per the South African Expanded Programme on Immunisation (EPI-SA) schedule. Proportions, medians, inter-quartile ranges (IQR) and regression were used to obtain the prevalence, time-at-risk, and risk factors for delayed immunisation. A total of 652 /709 (91.9%) participants were eligible. Immunisation coverage declined with age from 94.9% (95% CI 92.9–96.4) at birth to 72.0% (95% CI 65.7–77.6) at 18 months. The highest delay in the uptake of vaccine doses was observed among the 3 rd dose of the DTP vaccine [163 (34.6% (95% CI 30.3–39.1)], while the lowest was seen among BCG [40 (6.5% (95% CI 4.7–8.8)]. The longest median time-at-risk of VPDs was among the 2 nd dose of the measles vaccine [12.9 (IQR 6.7–38.6) weeks] and the lowest was OPV birth dose [IQR 6.3 (5.3–9.1) weeks]. Low and upper-middle socio-economic quartiles were associated with delayed uptake of vaccine doses. Delayed vaccination increases the time of susceptibility to VPDs during infancy and childhood. There is a need to develop strategies aimed at mitigating factors associated with delay in uptake of routine childhood vaccines in the Western Cape. Mitigation strategies should provide vaccine education and mobile reminder systems. Education about timely vaccine uptake will aid in the provision of informed council from healthcare providers to caregivers. Multiple reminder systems could cater for low network coverage areas and caregivers with busy schedules.

## Highlights

• Immunisation coverage has an inverse relationship with age, while delay in vaccine uptake has a direct relationship with age.
• High vaccine coverage rates do not translate to timely receipt of routine childhood vaccines.
• Time-at-risk for vaccine preventable diseases is positively associated with increasing immunisation age timepoints.
• Preschool attendance and having adult caregivers are protective against delaying vaccine uptake.
• Low and upper-middle socio-economic quartiles are associated with delayed uptake of routine childhood vaccines.

## Introduction

The World Health Organisation (WHO) first recommended the administration of routine childhood vaccines through the Expanded Programme on Immunisation (EPI) in 1974. Four decades on, this initiative has proven to be the most cost-effective public health strategy globally, reducing childhood morbidity, disability and death associated with vaccine preventable diseases (VPDs) [1], [2], [3], [4]. In 2019, the global immunisation coverage of the third dose of the diphtheria, tetanus, and pertussis vaccine (DTP-3) was 85%, with less than 19.7 million children (less than20%) deemed susceptible to VPDs [5]. Data received as of October 2020, show DTP-3 coverage to be 74% in Africa [6]. These figures are concerning as in 2019, WHO estimated that 5.2 million children under 5 years old had died from preventable diseases. Additionally, sub-Saharan Africa accounts for the highest number of preventable deaths (including VPDs) with 1 in 13 children dying before their fifth birthday [7].

In South Africa, routine childhood immunisation is reported to avert an estimated 2.5 million deaths annually [8]. The immunisation coverage for fully vaccinated under 1 year olds in 2017/8 was 77% [8].The Western Cape, Mpumalanga, Northern Cape, and Kwa-Zulu Natal provinces exceeded this national average [5]. In contrast, using the first measles dose as a proxy for under 1 year vaccine coverage, estimations by the WHO and the United Nations Children’s Fund (UNICEF) as of June 2020, show that immunisation coverage in South Africa has increased by only 2% from the previous report in 2017/8 [9]. As effective as vaccines have been proven to be, these low immunisation coverage figures in South Africa show that despite the provision of free vaccines, the efforts of the South African Expanded Programme on Immunisation (EPI-SA) by the South African Health System and government can potentially be undermined.

Outbreaks of VPDs such as measles and rubella in Gauteng, Kwa-Zulu Natal and the Western Cape provinces have been reported as recent as 2017 [10]. Additionally, cases of pertussis in the Western Cape were reported between October 2017 to January 2018 [11]. While these outbreaks are a cause for concern given that VPDs have the potential to cause serious morbidity and mortality, they also suggest that there might be an underlying problem since these outbreaks were documented in provinces that exceeded the national immunisation coverage rate [12], [13]. An improved understanding of the underlying determinants of outbreaks of VPDs despite optimal vaccination coverage rates could help inform evidence-based interventions. Moreover, understanding the barriers to vaccine uptake may assist in prioritising routine childhood vaccines amid global health emergencies, such as the current COVID-19 pandemic.

Immunisation coverage is defined as the proportion of people who receive vaccines at a certain age, regardless of the timing of administration [14]. While widely used to measure the performance of immunisation programs, immunisation coverage has its limitations [14], [15]. This is because immunisation coverage alone cannot inform on the level of adherence to the EPI schedule. For example, a study conducted using data from Soweto (Gauteng) and Pietermaritzburg (Kwa-Zulu Natal), in South Africa, found immunisation coverage at the sites to be 93.9% and 90.6% respectively. Despite this, it was also reported that 32.2% and 25.2% of the study participants delayed vaccine uptake respectively [16]. There is currently no consensus for how immunisation timeliness should be defined. However, previous studies conducted in low- and middle- income countries (LMIC) have described immunisation timeliness to be a) the receipt of vaccines at recommended ages and intervals, b) the interval, accessibility and the specificity of the vaccine, c) up-to-date immunisation at a certain threshold, and d) immunisation administration within a certain time in relation to the recommended age for immunisation [14], [17], [18]. Evidently, timeliness is not synonymous with coverage, but instead the two measures can be used together to get a population estimate for immunisation timing and coverage [14]. This distinction is important more so because lack of immunisation timeliness can potentially be a barrier to full immunisation coverage.

Immunisation coverage is related to the receipt or non-receipt of immunisations. While immunisation timeliness addresses the analytical questions such as whether immunisations were received on time, early, or delayed. Delays in immunisations are associated with inadequate developments of immune protection, which predisposes to the acquisition of VPDs [17]. The lack of a standard definition of delayed immunisation contributes to the complexity of quantifying delay and determining reasons for delay [18]. Despite the lack of a standard definition, generally delay in immunisations based on studies conducted in LMICs, has been described as ≥ 4 weeks (28 days) deviation from the EPI schedule [20], [21], [22], [23].

To mitigate the potential detrimental impact of delayed uptake of routine childhood vaccines, catch up immunisations are necessary to protect populations who missed or delayed immunisations. These strategies not only give individuals another chance of protection from VPDs, but population-based catch-up strategies can also reach areas of political conflict [24]. Essentially, effective catch-up vaccination programs should be informed by evidence on vaccine timeliness. Unfortunately, such evidence from South Africa is currently scarce.

This study sought to assess the timeliness of age-specific routine immunisation in children presenting with respiratory tract infections in the City of Cape Town, Western Cape Province, South Africa. Primarily, the study set to describe the proportion of children with delayed timeliness of age-specific routine immunisation and to describe the degree of this delay. As a secondary outcome, the study intended to investigate for risk factors that lead to children not receiving age-specific immunisations on time.

## Methods

### Study design

This study retrospectively analysed data collected during a parent prospective study that was conducted between 2012 and 2016. The parent study investigated the incidence and risk factors for pertussis in South African children less than 13 years of age. The children presented to four health facilities (i.e. Red Cross War Memorial Hospital, Mitchells Plain Day Hospital, Eastridge Clinic and Athlone - Silvertown Clinic) with mild to severe respiratory tract infections [25].

### Sample size

We estimated that a minimum of 500 children’s medical records of the original database would meet the inclusion criteria. This would give a sample size sufficient to give precisions within 5% of all possible estimates of the primary outcome between 20% and 50%, which we deemed to be acceptable.

### Selection of participants

The study included all participants from the original database for whom immunisation information as noted in the handheld immunisation record, the Road to Health Card (RTHC) was available. The information had to include the date each dose of the recommended vaccines was received. In addition, the participants’ Case Report Forms (CRFs) had to contain sufficient data to assess for potential risk factors.

Variables such as immunisation history, socio- demographic and economic data (e.g., participant age, care-giver socioeconomic status, care-giver education levels) were retrieved from the research database. Socio-economic status was categorized into inter-quartile ranges (IQRs) based on a validated weighted composite score that included asset ownership, employment, and education [26].

### Definitions

Eligibility at each time point was described as participants who were old enough to receive vaccines as recommended. Up-to-date was described as receiving all the recommended vaccines at the recommended age time points. On-time uptake of vaccines was described as vaccine doses received less than 5 days before and not longer than 28 days after the recommended age as per the EPI-SA schedule [14]. Early uptake of vaccine doses was defined as anything received earlier than 4 days before the recommended age [14]. Delayed uptake of vaccine dose was defined as receiving vaccines more than 28 days after the recommended schedule age [27].

### Exposures and outcomes

The primary outcome of interest was the receipt of a vaccine dose later than 28 days of that recommended for age as per the EPI-SA schedule (birth doses are no exception). To evaluate timeliness, proportions of participants receiving and not receiving age-specific immunisations, at the recommended time were described for each vaccine. Timeliness in immunisation was calculated as the difference in the expected date and the actual date of receipt of the age-specific immunisation. Duration of delay was described for those who did not receive timely immunisations. The Bacillus Calmette–Guérin (BCG) birth dose was used as a reference to compare the median duration in delays with age. BCG is expected to have the lowest delay in uptake, and therefore when used as a reference, presents an opportunity to depict trends (increase/decrease) for delays with age.

### Data analysis

Data were analysed using R version 3.5.1 (2018–07-02) and R studio version 1.3.1073 [28]. Where hypothesis testing was done, significance level was set at a two-tailed P less than 0.05. Demographic characteristics extracted from CRFs and RTHCs were tabulated to provide a background description of the study population. Continuous numerical data were described using medians and interquartile ranges (IQR). Proportions were depicted as percentages to describe categorical variables. Differences in distribution of continuous data were assessed using Mann-Whitney-U tests. Associations between two categorical variables were assessed using Chi-square or Fisher’s exact tests as appropriate.

Factors associated with delayed immunisation for each vaccine was evaluated using logistic regression using the binary outcome of Yes/No (Delayed/On time). All covariates in univariable analyses with a p-value of 0.05 were included in multivariable analyses.

### Ethical considerations

The study was approved by the University of Cape Town Faculty of Health Sciences Human Research Ethics Committee [HREC 027/2020].

## Results

### Demographic characteristics of the study population

**Fig. 1:**
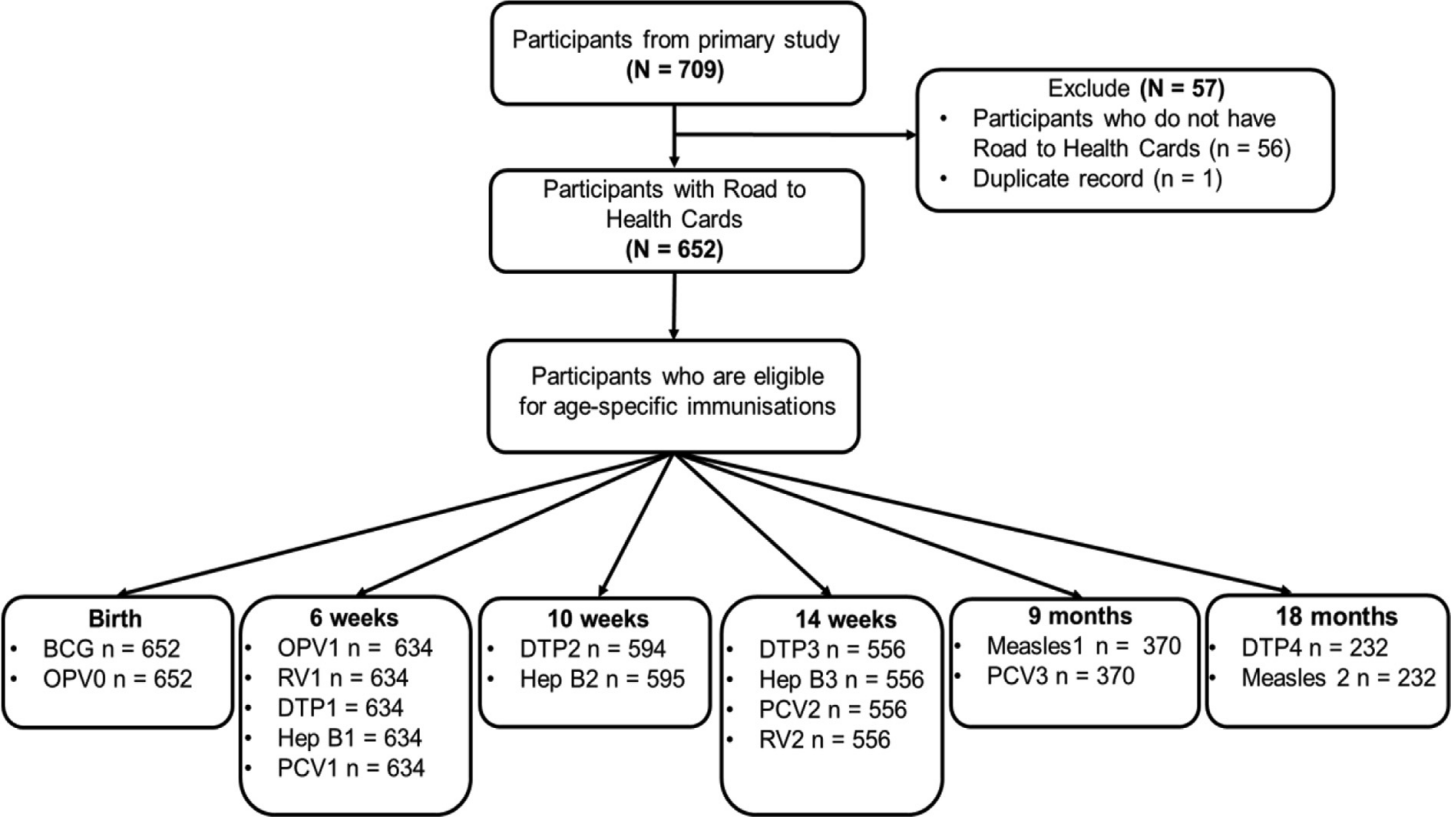
Flow diagram of participants included in the study. BCG – Bacillus Calmette- Guérin, OPV0 – birth dose Oral polio vaccine, OPV1 – 1st dose Oral polio vaccine, RV1 – 1st dose Rotavirus, DTP1 – 1st dose Diphtheria/Tetanus, Pertussis, Inactivated Poliomyelitis vaccine and Haemophilus Influenzae Type B (DTP/IPV/HiB), Hep B1 – ^1st^ dose Hepatitis B, PCV1 – 1st dose Pneumococcal Conjugate vaccine, DTP2 − 2nd dose DTP/IPV/HiB, Hep B2 – 2nd dose Hepatitis B, DTP3 − 3rd dose DTP/IPV/HiB, Hep B3 – 3rd dose Hepatitis B, PCV2 – 2nd dose Pneumococcal vaccine, RV2 – 2nd dose Rotavirus, Measles 1 – 1st dose Measles, PCV3 – 3rd dose Pneumococcal vaccine, DTP4 − 4th dose DTP/IPV/HiB, Measles 2 – 2nd dose Measles.

A total of 652/709 (92%) participants were eligible for analysis (Fig. 1). The median age of the included participants was 11 [IQR 4.5–28.0] months.

Most (360 [55.2%]) of the participants were male. Of the total 605 participants who were eligible to attend creche, 457 (75.5%) did not attend. Of the available 642 records in which participants divulged their racial background, 389 (60.6%) identified as Black, 247 (38.5%) as Coloured (mixed ancestry) and 6 (0.9%) as other races. Overall, 639 (98%) of caregivers were mothers, with a median age of 28 [IQR 24–34] years. A total of 622 (95.4%) caregivers reported having attained a basic level of education, with grade 11 [IQR 10–12] being the median highest grade reached (Table 1).

**Table 1:** Participant and caregiver baseline characteristics (N = 652).

Characteristic	n (%)
*Participant demographics*	
**Sex (n = 652)**	
Female	292 (44.8)
Male	360 (55.2)
**Race (n = 642) ^α^**	
Black	389 (60.6)
Coloured/Mixed ancestry	247 (38.5)
Other	6 (0.9)
**Creche ^¥^ (n = 605) ^α^**	
No	457 (75.5)
Yes	145 (24.0)
Unknown	3 (0.5)
*Caregiver demographics*	
**Relationship with child (n = 652)**	
Mother	639 (98.0)
Other	13 (2.0)
**Education (n = 625) ^α^**	
Higher education	21 (3.2)
Basic education	622 (95.4)
No school	1 (0.2)
Unknown	8 (1.2)
**Socio-economic IQR (n = 547) ^α^**	
Low	129 (23.6)
Lower-middle	53 (9.7)
Upper-middle	285 (52.1)
High	80 (14.6)

α – Missing data; ¥ – Preschool, Kindergarten, Nursery

### Immunisation coverage and timing of vaccine uptake

The findings on vaccine coverage and timeliness of immunisation within the study population is reported for each of the age time-points indicated in the EPI-SA (Table 2).

**Table 2:** Immunisation coverage and delay and duration time at risk of Vaccine Preventable Diseases.

Timepoints	Immunisation	Eligible n (%)	Coverage n (%)	Delay in weeks Median [IQR]
**Birth**	BCG	652 (1 0 0)	619 (94.9)	6.6 [5.4–9.1]
	OPV0	652 (1 0 0)	616 (94.5)	6.3 [5.3–9.1]
**6 weeks**	OPV 1	634 (97.2)	570 (89.9)	9.0 [6.3–18.3]
	Rotavirus 1	634 (97.2)	494 (77.9)	8.4 [5.7–21.4]
	DTP/IPV/HiB 1	634 (97.2)	599 (94.5)	7.0 [4.7–11.2]
	Hepatitis B1	634 (97.2)	579 (91.3)	7.6 [5.9–13.8]
	PCV 1	634 (97.2)	546 (86.1)	8.0 [5.3–12.9]
**10 weeks**	DTP/IPV/HiB 2	594 (91.1)	537 (90.4)	7.6 [5.0–12.4]
	Hepatitis B2	595 (91.3)	533 (89.6)	8. 9 [5.29–14.3]
**14 weeks**	DTP/IPV/HiB 3	556 (85.3)	471 (84.7)	7.9 [5.3–17.1]
	Hepatitis B3	556 (85.3)	471(84.7)	7.9 [5.1–13.3]
	PCV 2	556 (85.3)	421 (75.7)	7.7 [5.3–19.9]
	Rotavirus 2	556 (85.3)	388 (69.8)	7.4 [5.1–19.3]
**9 months**	Measles 1	370 (56.7)	314 (84.9)	8.6 [5.5–25.3]
	PCV 3	370 (56.7)	242 (65.4)	7.3 [5.0–23.4]
**18 months**	DTP/IPV/HiB 4	232 (35.6)	167 (72.0)	10.9 [8.0–28.7]
	Measles 2	232 (35.6)	167 (72.0)	12.9 [6.7–38.6]

### Birth dose vaccination

**Fig. 2:**
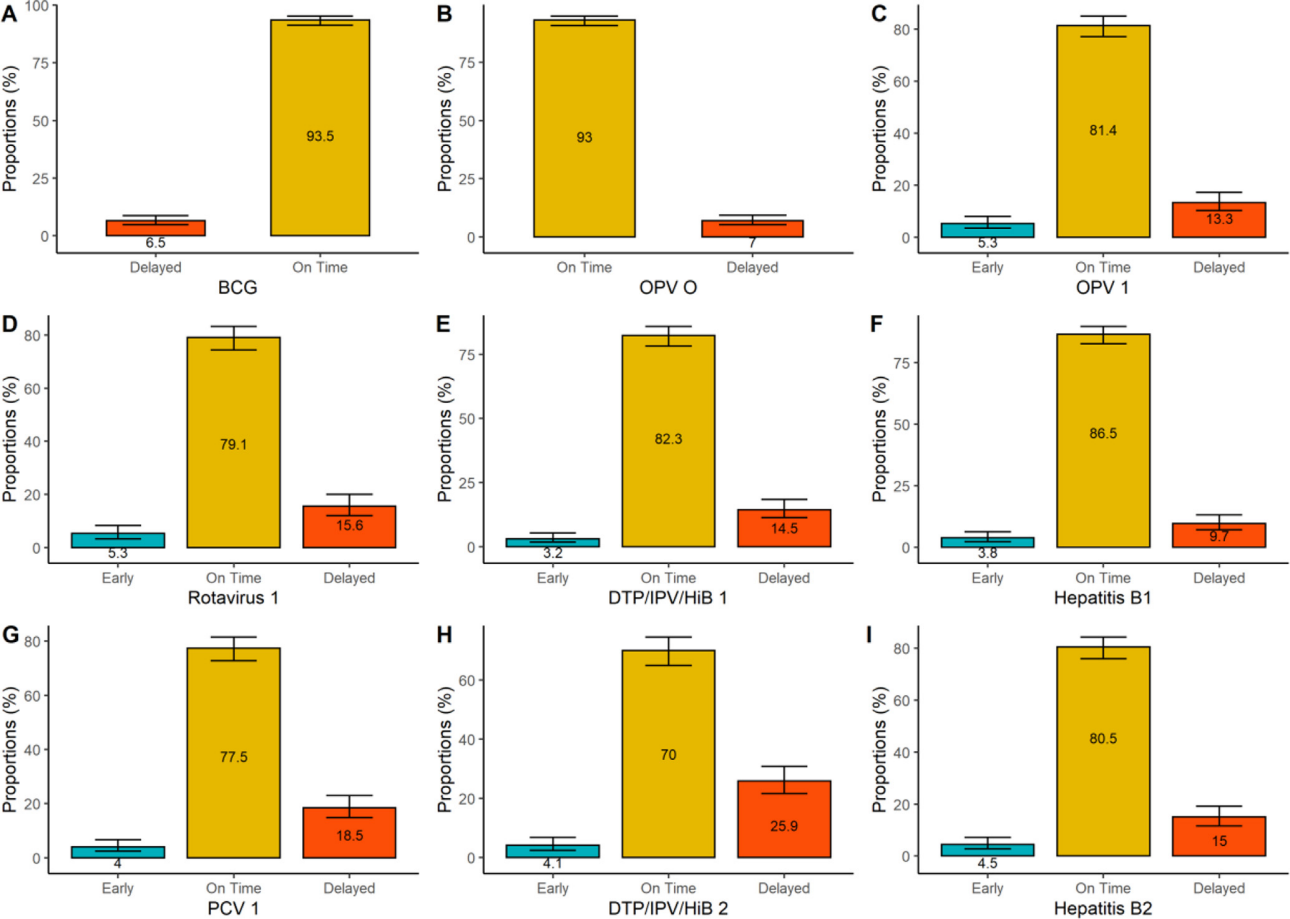
Timeliness of uptake of birth, 6- and 10-week vaccine doses. BCG (n = 619); OPV (n = 616); 1st doses of Rotavirus (n = 494), DTP/IPV/HiB (n = 599), Hepatitis B (n = 579), PCV (n = 546); 2nd doses of DTP/IPV/HiB (n = 537) and Hepatitis B (n = 533).

Vaccination with BCG vaccine was received by 619 (94.9%) of the 652 participants. Of the 619 participants who received BCG, 40 (6.5%) delayed uptake. The oral polio vaccine (OPV) birth dose was received by 616 (94.5%) of the study participants. Of the 616 participants who received OPV, 43 (7.0%) had delayed uptake (Fig. 2A and B).

### Vaccination at 6, 10 and 14 weeks

The 1st dose of DTP/IPV/HiB (combined vaccine against diphtheria, tetanus, pertussis, polio, and Haemophilus influenzae type B vaccine) was received by 599/634 (94.5%); the1^st^ dose of hepatitis B vaccine was received by 579/634 (91.3%), and the 1st dose of PCV was received by 546/634 (86.1%) (Table 2). Amongst those who received the 1st dose of DTP/IPV/HiB vaccine, 87 (14.5%) delayed uptake, 493 (82.3%) were on time, and 19 (3.2%) received the vaccine earlier than scheduled. A total of 56 (9.7%) delayed uptake for the 1st dose of hepatitis B vaccine, 501 (86.5%) of the participants’ uptake was on time, while 22 (3.8%) received their doses earlier than scheduled. Uptake of the 1st dose of the pneumococcal conjugate vaccine (PCV) was delayed in 101 (18.5%) of participants, while 423 (77.5%) and 22 (4%) of vaccine uptake was on time and early, respectively. Amongst all doses given at 6-weeks, the 1st dose of PCV had the highest proportion of participants who delayed uptake. The proportion of delayed participants for the 1st dose of OPV increased by 6.3% from the OPV dose given at birth. Early uptake of vaccine doses was observed in all vaccines at the 6-week age timepoints. The highest early uptake was among the 1st dose of OPV, and the Rotavirus vaccine doses (26 [5.3%]) (Fig. 2C-G).

Immunisation coverage of the 2nd dose of DTP/IPV/HiB and hepatitis B vaccines was 537/594 (90.4%) and 533/595 (89.6%), respectively (Table 2). Delayed uptake for the 2nd dose of DTP/IPV/HiB and hepatitis B vaccines was observed in 139 (25.9%) and 80 (15%) of participants, respectively. While 362 (70%) and 429 (80.5%) of participants had timely uptake. In addition to the decline in immunisation coverage from the 6-week to 10-week age timepoints, there was also an 11.4% and 5.3% increase in delay in uptake for the 2nd DTP/IPV/HiB and hepatitis B vaccine doses respectively, compared to the 1st doses. Early uptake of vaccine doses was observed for 22 (4.1%) participants for the 2nd dose of DTP/IPV/HiB and 24 (4.5%) participants for the 2nd dose of hepatitis B (Fig. 2 H and I).

**Fig. 3:**
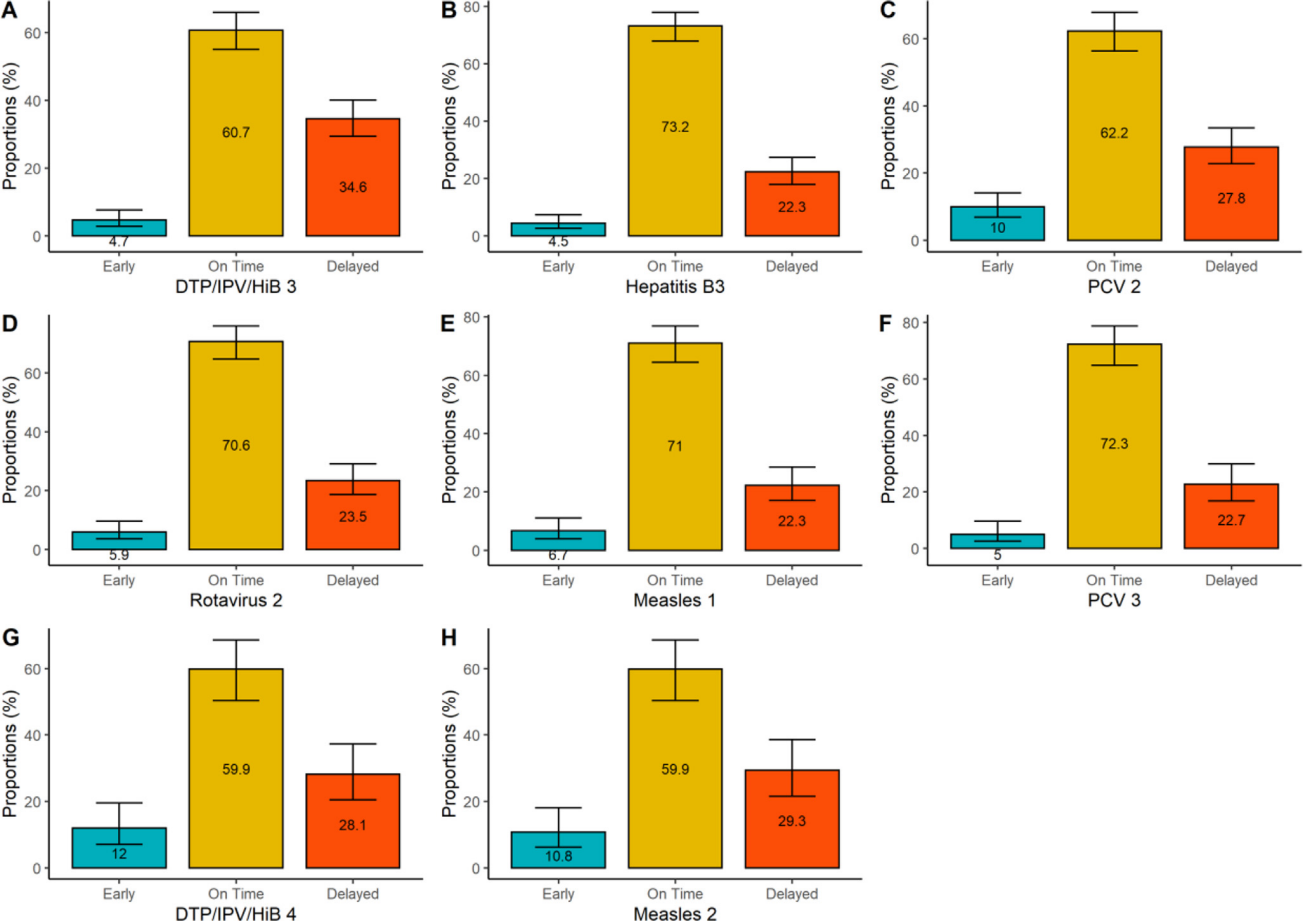
Timeliness of uptake for the 14 weeks, 9- and 18-months vaccine doses. 3rd doses of DTP/IPV/HiB (n = 471), and hepatitis B (n = 471), 2nd doses of PCV (n = 421), and Rotavirus (n = 388); 1st dose of Measles (n = 314), 3rd dose of PCV (n = 242); 4th dose of DTP/IPV/HiB (n = 167), 2nd dose of Measles (n = 167).

The 3rd dose of the DTP/IPV/HiB vaccine was received by 471/556 (84.7%) participants. Of those, 163 (34.6%) delayed vaccine uptake, while 286 (60.7%) had timely uptake. The 3rd dose of hepatitis B vaccine was received by 471/556 (84.7%), and of those, 105 (22.3%) delayed uptake, while 345 (73.2%) of participants had timely uptake. Immunisation coverage for the 2nd dose of PCV was 421/556 (75.7%). Amongst those 117 (27.8%) delayed uptake, while 262 (62.2%) had timely uptake of doses, as recommended. Regarding the vaccine doses administered at 14 weeks, the 3rd dose of hepatitis had the lowest proportion of participants with delayed vaccine uptake, while the 3rd dose of DTP/IPV/HiB vaccine had the highest proportion of participants who delayed vaccine uptake. When the timeliness of uptake of the vaccines administered at the 14-weeks timepoint was compared to their 10-week doses, there was an 8.7% increase for the 2nd dose of DTP/IPV/HiB vaccine, 7.3% increase for the 3rd hepatitis B vaccine dose, 9.3% increase for the 2nd PCV dose and 7.9% increase for the 2nd Rotavirus vaccine dose in delaying vaccine uptake. The 2nd dose of PCV had the highest (42 [10%]) early vaccine uptake in the 14-week age timepoint (Fig. 3A-D).

### Vaccination at 9 and 18 months

The 1st dose of measles vaccine was received by 314/370 (84.9%). Amongst those 70 (22.3%) delayed vaccine uptake, while 223 (71%) had timely uptake of the vaccine. The 3rd dose of PCV was received by 242/370 (65.4%) and of those, 55 (22.7%) of participants delayed uptake, while 175 (72.3%) had timely 3rd dose PCV vaccine uptake. While coverage of the 3rd dose of PCV decreased by 10.3% between the 2nd and 3rd doses, delayed uptake reduced by 5.1% from the 14 weeks timepoint. The 1st dose of the measles vaccine had 21 (6.7%) and the 3rd dose of PCV had 12 (5%) of participants who had early uptake of vaccines (Fig. 3E and F).

The 2nd dose of measles and the 4th dose of DTP/IPV/HiB vaccines were both received by 167/232 (72.0%) of participants. Delayed uptake among the measles and the DTP/IPV/HiB doses was observed in 49 (29.3%) and 47 (28.1%) of participants, respectively. Both 2nd dose of measles and the 4th dose of the DTP/IPV/HiB vaccines had 100 (59.9%) of participants who received timely uptake of vaccines. A reduction of 12.9% for the measles dose from the 9-month age timepoint and a 12.7% reduction for the DTP/IPV/Hib dose from the 14-week age timepoint in immunisation coverage was observed for both vaccines recommended for administration at 18 months, but the delay in uptake of the 4th dose of DTP/IPV/HiB reduced by 6.5% compared to the 3rd dose of DTP/IPV/HiB, while the delay in uptake of the 2nd dose of measles vaccine increased by 7% from 1st dose. Of all the recommended age timepoints, the 18-months age timepoint had the highest early uptake of vaccines. Early uptake was observed in 18 (12%) participants for the measles vaccine and 20 (10%) participants for the DTP/IPV/HiB vaccine (Fig. 3G and H).

### Duration of delay in age-specific immunisations

The degree of delayed age-specific routine immunisation was described using medians and inter-quartile ranges (IQR).

### Vaccinations at birth

The median delay in uptake of the BCG vaccine was 6.6 [IQR 5.4 – 9.1] weeks, while the OPV birth dose had a median delay in uptake of 6.3 [IQR 5.3 – 9.1] weeks. The median delay in uptake of the OPV birth vaccine dose was not significantly different to BCG (p = 0.97).

### Vaccinations at 6, 10 and 14 weeks

The median duration of delay in uptake of DTP/IPV/HiB vaccine doses increased with increasing age. The median duration of delay in uptake of the 1st, 2nd, and 3rd doses of the DTP/IPV/HiB vaccine administered at 6, 10 and 14 weeks was 7.0 [IQRs 4.7 – 11.2], 7.6 [IQRs 5.0 – 12.4] and 7.9 [IQRs 5.3 – 17.1] weeks, respectively (Table 2).

**Fig. 4:**
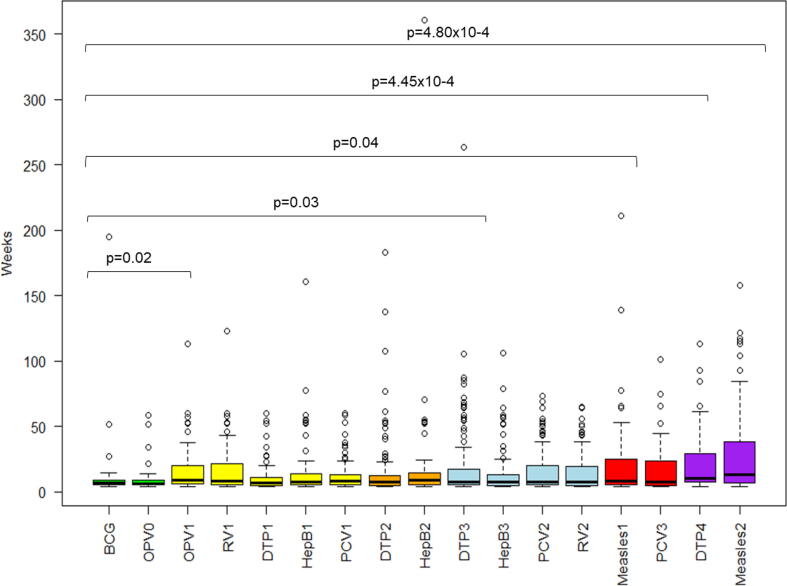
Change in median duration of delay in uptake of routine vaccines overtime. Green – birth doses, yellow – 6 weeks doses, orange – 10 weeks dose, blue – 14 weeks doses, red – 9 months doses, purple – 18 months doses.

The median duration of delay in uptake of the hepatitis B vaccine increased from 7.6 [IQR 5.9 – 13.8] weeks for the 1st dose to 8.9 [IQR 5.29 – 14.3] weeks for the 2nd dose. The median duration of delay of the 3rd dose of hepatitis B vaccine decreased to 7.9 [IQR 5.1 – 13.3] weeks. The median differences for all hepatitis B vaccine doses compared to BCG vaccine were not significant (Fig. 4).

The median duration of delay amongst the 1st and 2nd doses of the PCV vaccines given at 6 and 10 weeks declined with increasing age. The median duration of delay in the 1st dose of PCV was 8.0 [IQR 5.3 – 12.9] weeks, while the median duration of delay for the 2nd dose of PCV was 7.7 [IQR 5.3 – 19.9] weeks (Table 2). However, the median duration of delay between 1st and 2nd doses of the PCV vaccines were not significantly different to BCG at baseline (Fig. 4).

### Vaccinations at 9 and 18 months

The median duration of delay for the 9 months measles vaccine dose was 8.6 [IQR 5.5 – 25.3] compared to 12.9 [IQRs 6.7 – 38.8] weeks at 18 months; p = 0.07. (Fig. 4).

The median duration of delay for the 3rd dose of PCV at 9 months further declined from the 14 weeks age timepoint from 7.7 [IQR 5.3 – 19.9] to 7.3 [IQR 5.0 – 23.4] weeks (Table 2). There were no observed differences in the median duration of delay between the 2nd and 3rd doses of the PCV compared to BCG (Fig. 4).

The 4th dose of the DTP/IPV/HiB vaccine had a median duration of delay that increased to 10.9 [IQR 8.0 – 28.7] weeks from that of the 3rd dose of the DTP/IPV/HiB vaccine which was 7.9 [IQR 5.3 – 17.1] weeks. (Fig. 4).

### Risk factors associated with delayed immunisations

To determine which demographic and socio-economic factors are associated with delayed immunisations, logistic regressions analyses included sex, creche (preschool) attendance, caregiver age, education, and socio-economic status (SES) as covariates. Where data on these variables were limited or not available, these were excluded from the analysis (Table 1). Caregivers’ age was used as either a binary variable (adult/adolescent) or as a continuous variable, depending on the availability of data.

### Vaccinations at birth

None of the factors assessed were found to be statistically associated with the delay in uptake of the BCG and OPV vaccine doses (Table 3).

**Table 3:** Logistic Regression estimates.

Dose	Variable		Delayed n (%)	Univariable OR (95 % CI)	Multivariable OR (95% CI)
**BCG**	Sex	Male	40 (6.5)	0.52 (0.27–1.00)	
	Creche*	Yes		0.60 (0.24–1.30)	0.74 (0.26–1.76)
	Caregiver age*			0.98 (0.94–1.03)	1.00 (0.95–1.05)
	Caregiver age category	Adult		0.86 (0.24–5.49)	
	Education*	Higher		0.76 (0.04–3.79)	1.01 (0.05–6.08)
	Highest grade			1. 07 (0.86–1.38)	
	SES*	Low		0.99 (0.32–3.38)	1.06 (0.32–3.84)
		Lower-middle		0.57 (0.08–2.75)	0.64 (0.08–3.27)
		Upper-middle		1.06 (0.41–3.30)	0.99 (0.36–3.26)
**OPV 0**	Sex	Male	43 (6.98)	0.60 (0.32–1.12)	
	Creche*	Yes		0.88 (0.40–1.77)	1.11 (0.45–2.47)
	Caregiver age*			0.98 (0.94–1.02)	
	Caregiver age category	Adult		0.59 (0.20–2.55)	
	Education*	Higher		1.50 (0.23–5.46)	1.80 (0.25–7.89)
	Highest grade			1.15 (0.92–1.49)	
	SES*	Low		0.67 (0.23–1.99)	0.79 (0.26–2.51)
		Lower-middle		0.20 (0.01–1.18)	0.23 (0.01–1.41)
		Upper-middle		0.79 (0.33–2.07)	0.78 (0.31–2.21)
**OPV 1**	Sex	Male	76 (13.3)	1.42 (0.86–2.36)	
	Creche*	Yes		0.59 (0.31–1.07)	0.75 (0.38–1.40)
	Caregiver age			1.01 (0.98–1.04)	
	Caregiver age category	Adult		0.83 (0.30–2.91)	1.09 (0.35–4.80)
	Education*	Higher		1.08 (0.25–3.32)	1.24 (0.25–4.44)
	Highest grade			0.95 (0.82–1.12)	
	SES*	Low		0.75 (0.31–1.81)	0.84 (0.34–2.13)
		Lower-middle		0.85 (0.29–2.36)	0.98 (0.32–2.90)
		Upper-middle		0.86 (0.42–1.87)	0.95 (0.44–2.18)
**Rotavirus 1**	Sex	Male	77 (15.6)	0.99 (0.61–1.63)	
	Creche*	Yes		0.78 (0.43–1.42)	1.10 (0.57–2.03)
	Caregiver age			0.99 (0.95–1.02)	
	Caregiver age category	Adult		**0.29 (0.12**–**0.72)**	**0.33 (0.13**–**0.94)**
	Education*	Higher		0.35 (0.02–1.80)	0.36 (0.02–2.04)
	Highest grade			1.06 (0.89–1.29)	
	SES*	Low		0.45 (0.18–1.12)	0.41 (0.16–1.06)
		Lower-middle		0.54 (0.13–1.73)	0.47 (0.11–1.54)
		Upper-middle		0.82 (0.41–1.75)	0.74 (36–1.61)
**DTP/IPV/HIB 1**	Sex	Male	87 (14.5)	1.23 (0.77–1.96)	
	Creche*	Yes		1.40 (0.85–2.27)	1.52 (0.87–2.61)
	Caregiver age			1.02 (0.99–1.04)	
	Caregiver age category			0.81 (0.32–2.47)	0.85 (0.31–3.03)
	Education*	Higher		0.62 (0.10–2.20)	0.34 (0.02–1.82)
	Highest grade			1.03 (0.88–1.21)	
	SES*	Low		0.75 (0.33–1.76)	0.69 (0.27–1.57)
		Lower-middle		0.83 (0.29–2.25)	0.68 (0.23–1.92)
		Upper-middle		0.10 (0.51–2.08)	0.89 (0.44–1.89)
**Hepatitis B1**	Sex	Male	56 (9.67)	0.93 (0.54–1.63)	
	Race	Coloured		0.82 (0.45–1.46)	
	Creche*	Yes		**0.49 (0.22**–**0.98)**	0.59 (0.23–1.31)
	Caregiver age			1.03 (0.99–1.06)	
	Caregiver age category	Adult		0.79 (0.26–3.44)	0.98 (0.27–6.32)
	Education*	Higher		1.72 (0.39–5.36)	1.82 (0.36–6.98)
	Highest grade			0.93 (0.78–1.12)	
	SES*	Low		0.51 (0.19–1.34)	0.62 (0.22–1.74)
		Lower-middle		0.58 (0.15–1.89)	0.55 (0.11–2.09)
		Upper-middle		0.57 (0.26–1.32)	0.64 (0.28–1.60)
**PCV 1**	Sex	Male	101 (18.5)	1.13 (0.73–1.76)	
	Creche*	Yes		1.07 (0.63–1.76)	1.23 (0.69–2.15)
	Caregiver age			1.00 (0.98–1.04)	
	Caregiver age category	Adult		0.83 (0.34–2.31)	0.93 (0.35–2.90)
	Education*	Higher		0.24 (0.01–1.18)	0.28 (0.02–1.55)
	Highest grade			1.00 (0.86–1.17)	
	SES*	Low		1.11 (0.51–2.50)	0.98 (0.44–2.26)
		Lower-middle		1.16 (0.41–3.12)	0.84 (0.28–2.39)
		Upper-middle		1.21 (0.62–2.53)	1.10 (0.55–2.34)
**DTP/IPV/HIB 2**	Sex	Male	139 (25.9)	1.19 (0.81–1.77)	
	Creche*	Yes		0.86 (0.55–1.32)	0.95 (0.58–1.54)
	Caregiver age			0.99 (0.97–1.02)	
	Caregiver age category	Adult		0.79 (0.32–2.10)	0.82 (0.30–2.45)
	Education*	Higher		0.71 (0.20–2.01)	0.69 (0.15–2.42)
	Highest grade			0.89 (0.78–1.02)	
	SES*	Low		1.26 (0.63–2.57)	1.12 (0.54–2.37)
		Lower-middle		0.62 (0.22–1.60)	0.63 (0.21–1.68)
		Upper-middle		1.47 (0.80–2.79)	1.48 (0.77–2.81)
**Hepatitis B2**	Sex	Male	80 (15)	1.26 (0.78–2.05)	
	Creche*	Yes		**0.52 (0.28**–**0.93)**	0.68 (0.33–1.32)
	Caregiver age			0.99 (0.97–1.03)	
	Caregiver age category	Adult		0.51 (0.20–1.45)	0.60 (0.20–2.19)
	Education*	Higher		1.08 (0.25–3.35)	1.40 (0.29–5.25)
	Highest grade			0.87 (0.75–1.00)	
	SES*	Low		0.89 (0.40–2.04)	0.94 (0.40–2.28)
		Lower-middle		0.33 (0.07–1.11)	0.40 (0.08–1.43)
		Upper-middle		0.72 (0.35–1.56)	0.73 (0.34–1.67)
**DTP/IPV/HIB 3**	Sex	Male	163 (34.6)	1.37 (0.93–2.03)	
	Creche*	Yes		0.81 (0.53–1.23)	0.82 (0.50–1.35)
	Caregiver age			1.00 (0.98–1.03)	
	Caregiver age category	Adult		0.70 (0.27–1.87)	0.50 (0.15–1.55)
	Education*	Higher		1.12 (0.39–3.22)	1.13 (0.27–4.31)
	Highest grade			0.89 (0.78–1.02)	
	SES*	Low		0.97 (0.47–2.02)	0.93 (0.44–2.03)
		Lower-middle		0.61 (0.24–1.51)	0.52 (0.18–1.38)
		Upper-middle		1.24 (0.66–2.41)	1.34 (0.69–2.66)
**Hepatitis B3**	Sex	Male	105 (22.3)	1.34 (0.86–2.10)	
	Creche*	Yes		**0.39 (0.22**–**0.66)**	**0.48 (0.22**–**0.86)**
	Caregiver age			1.01 (0.99–1.05)	
	Caregiver age category	Adult		0.42 (0.16–1.18)	0.32 (0.10–1.06)
	Education	Higher		0.59 (0.09–2.24)	
	Highest grade*			0.85 (0.74–0.98)	0.84 (0.71–1.00)
	SES*	Low		1.00 (0.46–2.23)	0.66 (0.26–1.65)
		Lower-middle		0.44 (0.13–1.29)	0.30 (0.06–1.07)
		Upper-middle		0.86 (0.43–1.81)	0.71 (0.33–1.60)
**PCV 2**	Sex	Male	117 (27.8)	1.01 (0.66–1.58)	
	Creche*	Yes		**0.56 (0.32**–**0.94)**	0.58 (0.30–1.10)
	Caregiver age			1.02 (0.99–1.05)	
	Caregiver age category	Adult		0.55 (0.20–1.59)	
	Education	Higher		0.66 (0.15–2.22)	
	Highest grade*			0.78 (0.66–0.92)	0.81 (0.66–1.00)
	SES*	Low		2.47 (1.06–6.15)	1.75 (0.66–4.90)
		Lower-middle		1.02 (0.31–3.23)	1.20 (0.35–4.02)
		Upper-middle		2.36 (1.10–5.52)	2.25 (0.99–5.64)
**Rotavirus 2**	Sex	Male	91 (23.5)	0.84 (0.45–1.59)	
	Creche*	Yes		0.55 (0.23–1.17)	0.79 (0.32–1.80)
	Caregiver age			0.96 (0.92–1.00)	
	Caregiver age category *	Adult		**0.30 (0.10**–**0.99)**	0.45 (0.12–2.17)
	Education*	Higher		0.62 (0.03–3.31)	0.74 (0.04–4.58)
	Highest grade			1.08 (0.87–1.39)	
	SES*	Low		0.44 (0.13–1.41)	0.44 (0.13–1.44)
		Lower-middle		0.40 (0.06–1.83)	0.40 (0.05–1.87)
		Upper-middle		0.75 (0.30–2.04)	0.76 (0.30–2.11)
**Measles 1**	Sex	Male	70 (22.3)	0.96 (0.56–1.65)	
	Creche*	Yes		0.78 (0.44–1.36)	0.99 (0.49–1.98)
	Caregiver age			1.01 (0.87–1.06)	
	Caregiver age category *	Adult		0.96 (0.22–6.67)	0.63 (0.10–4.97)
	Education	Higher		0.28 (0.02–1.47)	
	Highest grade*			0.86 (0.72–1.04)	0.91 (0.71–1.16)
	SES*	Low		3.04 (0.98–11.54)	2.60 (0.64–13.36)
		Lower-middle		0.99 (0.18–4.88)	1.24 (0.20–7.55)
		Upper-middle		**3.90 (1.41**–**13.84)**	**4.39 (1.37**–**19.71)**
**PCV 3**	Sex	Male	55 (22.7)	0.94 (0.51–1.73)	
	Creche*	Yes		0.95 (0.49–1.81)	1.01 (0.42–2.34)
	Caregiver age			1.01 (0.97–1.05)	
	Caregiver age category *	Adult		2.30 (0.40–43.59)	1.51 (0.21–30.70)
	Education	Higher		1.06 (0.15–4.77)	
	Highest grade*			0.89 (0.72–1.11)	1.10 (0.81–1.53)
	SES*	Low		**3.00 (0.92**–**11.81)**	**5.09 (1.15**–**28.17)**
		Lower-middle		1.45 (0.26–7.47)	1.23 (0.15–8.55)
		Upper-middle		2.11 (0.72–7.76)	2.82 (0.83–13.12)
**DTP/IPV/HiB 4**	Sex	Male	47 (28.1)	0.72 (0.36–1.44)	
	Creche*	Yes		**0.48 (0.23**–**0.98)**	0.53 (0.19–1.37)
	Caregiver age*			1.02 (0.97–1.07)	0.10 (0.97–1.09)
	Education*	Higher		0.59 (0.09–2.56)	1.17 (0.14–8.15)
	Highest grade			1.08 (0.85–1.40)	
	SES*	Low		0.64 (0.17–2.30)	0.35 (0.19–2.93)
		Lower-middle		1.20 (0.30–4.79)	0.76 (0.42–9.35)
		Upper-middle		0.42 (0.13–1.33)	1.96 (0.14–1.55)
**Measles 2**	Sex	Male	49 (29.3)	0.82 (0.41–1.63)	
	Creche*	Yes		**0.49 (0.24**–**0.99)**	**0.37 (0.14**–**0.92)**
	Caregiver age*			1.02 (0.98–1.08)	1.05 (0.99–1.12)
	Education*	Higher		0.67 (0.06–3.02)	1.77 (0.18–17.78)
	Highest grade			1.08 (0.85–1.39)	
	SES*	Low		1.03 (0.26–4.29)	1.30 (0.30–5.88)
		Lower-middle		1.65 (0.37–7.70)	2.64 (0.52–14.42)
		Upper-middle		1.32 (0.41–4.75)	1.53 (0.45–5.86)

* Variables adjusted for in multivariable analysis

### Vaccinations at 6, 10 and 14 weeks

On average, those whose caregivers were adults had lower odds of delaying vaccine uptake, with 0.33 (CI 0.13 – 0.94) times the odds of delaying the 1st dose of rotavirus vaccine compared to those whose parents were adolescents (Table 3).

### Vaccinations at 9 and 18 months

An adjusted model for the 1st dose of the measles vaccine suggested a harmful association, where those of upper-middle SES compared to those of high SES had 4.39 (CI 1.37 – 19.71) times the odds of delaying the 1st dose of the measles vaccine. Low SES was a risk factor in delaying the 3rd dose of the PCV vaccine, with 5.09 (CI 1.15 – 28.17) times the odds compared to those of high SES (Table 3).

Attending creche was protective in delaying the 4th dose of DTP/IPV/HiB, but this protective effect was attenuated in the adjusted model. Creche attendance was protective for the 2nd dose of measles, compared to those who did not attend creche (Table 3).

## Discussion

This study found that proportions of children with delay in timely uptake of immunisations increases with age. Similarly, the median duration of delay increases with age. These trends are coupled with a decline in immunisation coverage and an increased probability of delaying uptake of subsequent doses when previous doses have been delayed. The highest vaccine coverage rates were seen amongst the birth doses, while the lowest was amongst the 9- and 18-months vaccine doses. Delay in receiving doses was independently associated with creche attendance, having an adult caregiver, and being in low and upper-middle socioeconomic quartiles.

Declining immunisation coverage with increasing age is not unique to this study. Prior studies have reported low immunisation coverage with vaccines given at 9- and 18- months and high immunisation coverage for the birth doses [29], [30]. During the 2015 global shortage in BCG vaccines, the Western Cape province had less than 50% of vials available to administer to new-borns [31]. It is highly likely that this shortage was associated with the observed delayed uptake of the BCG vaccines in our study population. To support this notion, we found a median of 6.6 [5.4 – 9.1] weeks delay in the uptake the BCG vaccine, which could be suggestive of catch-up vaccination campaigns conducted for those who were missed during the global shortage. Additionally, the minimum recommended time of 24 h for the receipt of the birth dose is a contributing factor to timely uptake of vaccines in that caregiver-child pairs visit health care facilities often post-partum and as a result, missed opportunities for birth doses are a rare occurrence [32], [33], [34]. In contrast, the low immunisation coverage for the 9- and 18-months vaccines could be a result of caregivers’ inability to adhere to the schedule due to busy work schedules and caregivers’ limited awareness about risk of VPDs at the 9- and 18-months immunisation age timepoints [35], [36]. The observed trend between the DTP/IPV/HiB doses administered at 6,10 and 14 weeks, where the delay in uptake of vaccines increased from 1st to the 3rd dose of the DTP/IPV/HiB vaccine was not unique to our study. A Gambian study reported similar findings stating that delaying on previous doses increases the chances of delaying for the subsequent doses [37]. Additionally, since the 3rd dose of the DTP/IPV/Hib vaccine serves as a national immunisation marker for coverage, and it can be used to assess the capacity of the health system to ensure that children return to receive all their scheduled doses. Given the sub-optimal coverage for the 3rd dose of the DTP/IPV/Hib vaccine found in this study which was below the 95% global target, it could be deduced that the performance of the EPI-SA, at least in the year 2016, was inadequate in controlling the disproportionate burden of VPDs in the Western Cape province [14].

The observed trends of increasing delay [Highest: DTP-3 34.6% (CI 30.3–39.1) and lowest BCG: 6.5% (CI 4.7–8.8)] in the uptake of immunisation and increased time at risk of VPD was not surprising. Several studies have found similar findings where significant increases in delayed uptake of vaccine doses with age were seen, especially for vaccines given after the first year of life [38], [39], [27], [40]. Due to the lack of appropriate variables or proxies in our dataset we could not assess for reasons for delayed presentation for immunisations, however drawing from similar settings, it is most likely that catch-up vaccinations are a result of the opportunistic presentation of caregiver-child pairs to health care facilities for other childhood sicknesses, leading to the administration of the appropriate catch-up vaccines by health care workers [38], [39], [40].

In this study, vaccine doses given at 9- and 18 months had the highest proportion of early uptake of vaccines. Due to the known high proportions of delay, early administration of measles has been observed in health care facilities as means to alleviate the delay in uptake of vaccines in some populations [7]. Prior research contradicts the early administration of measles since this approach has been reported to confer no protection [7], [15]. This is because early administration of the primary measles vaccine dose has been associated with suboptimal immunogenicity and the lack of compensation from the subsequent doses given at 9 months or older [19]. However, a recent systematic review and *meta*-analysis found that administering the primary measles vaccine dose before 9 months of age can elicit a good immune response in high-risk settings, and that a subsequent dose further increases vaccine effectiveness, rather than attenuate the immune response [19]. These findings of early vaccine administration are particularly concerning from the health systems perspective. It seems health care providers were more comfortable in administering doses earlier than scheduled to children beyond the first year of life, which could be considered as malpractice. Efforts to prevent these unrecommended practices by health care workers can include adequate training about the importance of adhering to the recommended schedule. The early administration of vaccines scheduled for the 18-months age timepoint also have the potential to give an underestimation of the delayed uptake of vaccines in populations, which can subsequently affect the planning of catch-up programs.

When we explored risk factors associated with the delay in uptake of scheduled vaccines, creche attendance was a common protective factor at the 18-months age timepoint. It is not surprising to see a protective effect from creche attendance, as the Department of Basic Education in South Africa requires parents or caregivers to provide up-to-date immunisation records as part of applications for admission to public or independent schools. Similarly, the Western Cape Education Department’s regulations requires up-to-date RTHCs before a child can be registered in any public school (independent schools in the province have their own admission rules) [24], [41]. This practice encourages parents to vaccinate their children. Having an adult caregiver was another protective factor against delayed uptake of the 1st dose of the rotavirus vaccine. This finding is similar to previous literature which finds that having an adult caregiver of ≥ 35 years of age is associated with lower odds of delaying uptake of primary immunisations [42]. Similar to our study, low SES IQR is a frequently cited strong risk factor for delaying uptake of vaccines, as low SES is largely coupled with low education levels, and thus limited knowledge about vaccines of risk of VPDs [16], [20], [43], [44]. It was surprising however, to find that upper-middle SES IQR was harmful towards delaying uptake of the 1st dose of the measles vaccine. We could not assess the reasons for this, although, it could be speculated that parents and caregivers in the upper-middle SES tend to delay or miss clinic visits due to busy work schedules [39], [45]. Future studies should assess reasons for delayed or missed vaccine uptake among populations within the upper-middle SES.

Education about timely vaccine uptake will aid in the provision of informed council from healthcare providers to – not only adult caregivers - but adolescent caregivers as well, with the aim to reduce delayed uptake of vaccine amongst those raised by adolescent caregivers. The health system and the EPI-SA should couple these interventions with effective mobile health strategies (i.e., mobile reminder systems). These reminder systems will particularly serve to remind those caregivers who delay uptake of vaccines because of busy work schedules. A recent South African multi-centre study reported the use of mobile reminders to effectively increase uptake of recommend maternal and childhood immunisations [46]. It is worth noting that in LMICs, mobile reminders have been reported to be effective only when caregivers receive two or more reminders [47]. It should be noted that alternative and complementary reminder systems may be required in settings with limited network coverage or access to mobile services.

Taken together, these findings suggest sub-optimal immunisation coverage and lack of immunisation timeliness in this population. The considerable increase in the delay in uptake of vaccines and the increased time at risk of VPDs is concerning as this may increase the pool of infants and children susceptible to VPDs. These findings emphasize the need of interventions targeting immunisation timeliness at infancy and early childhood.

This study has several strengths which are worth noting. Firstly, our study provides an update on the coverage and timeliness of routine childhood vaccines in the Western Cape province. Secondly, quantifying delay has the potential to aid in better understanding potential outbreaks of VPDs in the Western Cape province, thus informing about which age-groups should be of interest to immunisation program managers and policy makers. Limitations of this study include the use of RTHCs in verifying vaccination history which could be misleading in instances where the accuracy and quality of the data was compromised. This limitation emphasise the need for electronic immunisation records [48]. Finally, the geographical and participant focus of the study was restricted to a health facility-based population in the Western Cape province which may not be representative of all provinces in South Africa.

## Conclusion

This study underscores the distinction between immunisation coverage and timeliness as separate entities, which are both critical in understanding the effectiveness of routine childhood immunisation programmes in preventing the high burden of VPDs. Our findings of declining immunisation coverage, increasing delay and increased time-at-risk of VPDs with increasing age calls for immediate attention as the results have the potential to undermine the EPI-SA. Catch-up immunisation campaigns seem to be effective in minimising missed immunisation opportunities in the Western Cape province. However, more effort is required from the health system including immunisation service providers in firstly ensuring timely uptake to ensure optimal protection from VPDs and secondly, to ensure that catch-up immunisation services are readily available and accessible to caregivers. Effective surveillance and monitoring of immunisation programmes at all levels is required to achieve effective immunisation service delivery and uptake. In the current COVID-19 context, it will be important to understand how disruptions to immunisation services have further impacted timely uptake of routine childhood vaccines and potentially derailed existing VPD control efforts.

## Declaration of Competing Interest

The authors declare that they have no known competing financial interests or personal relationships that could have appeared to influence the work reported in this paper.
